# Patient satisfaction in a Moroccan emergency department

**DOI:** 10.1186/1755-7682-6-20

**Published:** 2013-05-04

**Authors:** Nada Damghi, Jihane Belayachi, Bouchra Armel, Aicha Zekraoui, Naoufel Madani, Khalid Abidi, Abdellatif Belabes Benchekroun, Amine Ali Zeggwagh, Redouane Abouqal

**Affiliations:** 1 Chirurgical Emergency Department, Ibn Sina University Hospital, Rabat 10000, Morocco; 2 Medical Emergency Department, Ibn Sina University Hospital, Rabat 10000, Morocco; 3 Medical Intensive Care Unit, Ibn Sina University Hospital, Rabat 10000, Morocco; 4 Laboratory of Biostatistics, Clincial, and Epidemiological Research, Faculté de Médecine et de Pharmacie - Université Mohamed V, Rabat 10000, Morocco

**Keywords:** Emergency care, Emergency department quality Study, Satisfaction

## Abstract

**Background:**

Measuring healthcare quality and improving patient satisfaction have become increasingly prevalent, especially among healthcare providers and purchasers of healthcare. Currently, research is interested to the satisfaction in several areas, and in various cultures. The aim of this study was; to confirm the reliability and validity of the Arabic version of the Emergency Department Quality Study (EDQS), to evaluate patient satisfaction with emergency care, and to determine associated factors with patient satisfaction.

**Methods:**

A survey of socio demographic, visit and health characteristics of patients, conducted in emergency department (ED) of a Moroccan University Hospital during 1 week in February 2009. The EDQS was performed with patients who were discharged from ED. The psychometric properties of the EDQS were tested. Factors influencing patient satisfaction were identified using ordinal logistic regression.

**Results:**

A total of 212 patients were enrolled. The Arabic version of the EDQS showed excellent reliability and validity. Sixty six percent of participants were satisfied with overall care, and 69.8% would return to our unit. The most patient-reported problems were about waiting time and test results. Variables associated with greater satisfaction with ED care were: emergent (OR: 0.15; 95% CI = 0.04-0.31; *P* < 0.001), or urgent patients (OR: 0.35; 95% CI = 0.15-0.86; *P* = 0.02) compared to non-urgent patients, and waiting time less than 15 min (OR: 0.41; 95% CI = 0.23-0.75; *P* = 0.003). Variables associated with lesser satisfaction were: distance patient’s home hospital ≤10Kilometers (OR: 2.64; 95% CI = 1.53-4.53; *P* < 0.001), weekday’s admissions (OR: 2.66; 95% CI = 1.32 to 5.34; *P* < 0.006), and educational level; with secondary (OR: 5.19; 95% CI = 2.04-13.21; *P* < 0.001) primary (OR: 3.04; 95% CI = 1.10-8.04; *P* = 0.03) and illiterate patients (OR: 2.53; 95% CI = 1.02-6.30; *P* = 0.03) were less satisfied compared to those with high educational level.

**Conclusion:**

Medical staff needs to consider different interactions between those predictive factors in order to develop some supportive tools.

## Background

Measuring healthcare quality and improving patients’ satisfaction have become increasingly prevalent, especially among healthcare providers and purchasers of healthcare, because consumers becomes more knowledgeable about healthcare [1]. Health care in developing countries has not traditionally focused on emergency medical care [2]. However, emergency care can make an important contribution to reduce avoidable deaths and disability in low-and middle-income countries, and in this realm, greater attention is needed [3,4].

Morocco has a total population of 31,285,174, gross national income per capita is $ 3.860. The health budget corresponds to 1.1 percent of gross domestic product and 5.5 percent of the central government budget [5]. Morocco has inadequate numbers of physicians (0.5 per 1,000 people) and hospital beds (1.0 per 1,000 people) and poor access to water (82% of the population) and sanitation (75% of the population). The health care system includes 122 hospitals, 2.400 health centers, and 4 university clinics, but they are poorly maintained with inadequate capacity to meet the demand for medical care [5]. Only 24.000 beds are available for 6 million patients seeking care each year, including 3 million emergency cases [5]. Morocco has two major health sectors, public and private, said to be complementary rather than competitive. Patients may choose whether to attend primary or secondary, public or private care. The majority of Moroccans in employment pay for health insurance, which covers most, but not all, of health expenses within the public and private sectors. The collective financing healthcare concerns only 41% of overall health expenditure. Only 5 million Moroccans have medical coverage, despite the social insurance system established for 40 years. The basic medical insurance is amongst the responses to deficit of social indicators in the field of health. In 2012, access to basic health care has been extended to poor by the implementation of a regime of medical assistance to economically disadvantaged patients (RAMED) and a compulsory health insurance.

The Emergency Department Quality Survey (EDQS) estimates satisfaction with overall care, willingness to return, and patient-reported problems to patient satisfaction [6]. A predictive model of patient satisfaction was previously created from the EDQS [6,7]. This model was validated by Sun et al. in patients who were discharged from ED [7]. Previous study concerning the measurement of patient satisfaction in a Moroccan acute medicine department has been carried by us [8] However; no studies of patients’ satisfaction with emergency care have been conducted in Morocco. The first objective of the present study was to confirm the reliability and validity of the Arabic version of the EDQS. The second objective was to evaluate patient satisfaction with emergency care in Morocco by using the EDQS; and to assess the determinants of patient satisfaction.

## Methods

This was a survey of patients conducted in Emergency Department (ED) of Rabat University Hospital during one week of February 2009. All patients admitted to ED on each day of the study, and aged more than 18 years were included. The study days were chosen based on the investigator's availability, and both weekdays and week-ends were included. The professional status of investigators was an emergency physician. Patients who did not have the mental capacity to fully understand and consent for the survey were excluded. A lack of mental capacity could be due to: Psychiatric disorder; Dementia; Confusion, drowsiness or unconsciousness because of an illness or the treatment; Substance misuse. Ibn Sina university hospital in Rabat is referral for habitants in Western-North Morocco, it is a 1028 bed tertiary – stage hospital that opened in 1955. The bed occupancy rate is of 76% to 85%. The hospital comprises 24 departments (12 surgical, 9 medicals, and 3 intensive care units). This hospital provides all major adult medical and surgical services except gynecology-obstetric, ophthalmology, otolaryngology, and oncology. The mean of ED visits per day is 176. The ED comprises two units (medical and chirurgical). Admission stay should not be greater than 72 hours. The medical staff is constituted by a senior doctor (emergency physicians with greater than 2 years experience in the unit) and 5 juniors (emergency physicians, and residents’ internal juniors with less than 2 years experience in the emergency unit). A patient can be accompanied by 2 members of his close relations who will stay in the waiting room until the end of the care. When an admission was necessary, the patient could also receive 2 visitors at fixed periods, for 3 hours per day. There are three others emergency department 40 km around our University Hospital. The study protocol was approved by the Moroccan Rabat University ethics committee, and informed consent was obtained from all participants.

The survey questionnaire includes the variables related to patient (demographic, socioeconomic, and health characteristics), and those related to patient – practitioner relationship. Demographic characteristics of patients included: age, gender, marital status (married, separated/single/widowed), residence (urban, rural); and distance from patient’s home to hospital (≤10 km, >10 km). Socioeconomic characteristics of patients included: educational level (high educational level, no education/primary/secondary level), monthly income (none, less than 180 euro, more than 180 euro); and health insurance status (yes, no). Health characteristics of patients measured at admission included: the level of the emergency (emergent, urgent, or non urgent); the day (weekdays, week-ends or holidays), the time of presentation (8–20 hours versus 20–8 hours). Characteristics related to the patient – practitioner relationship were: main diagnostic, the waiting time, and guidance of patients discharge or transfer). Waiting time was defined by the duration from the time patients registered in the emergency department to the time they were seen by a doctor [9-11].

**Figure 1:**
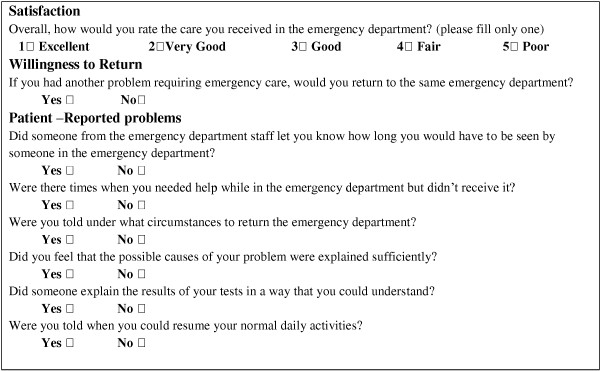
**EDQS (Emergency Department Quality Study)**^**5**^**.**

A predictive model of patient satisfaction was previously created from the EDQS [6,7]. This model was validated by Sun and al in the broader setting of all patients who were discharged from ED [8]. It estimates satisfaction with overall care, willingness to return, and patient-reported problems to patient satisfaction. Patient satisfaction with overall care was assessed on a 5 point likert scale (poor, fair, good, very good, excellent). Willingness to return was assessed on a binary yes-no scale. Six specific problems reported to be related to patient satisfaction have been studied with two modalitic binary answers for each question (yes or no) (Figure 1) [6]. Because no Arabic version of the EDQS was available, translation procedures followed by a transcultural adaptation were undertaken following international guidelines [12]. The following steps were used; in the first phase, the EDQS was translated by three bilingual (native Arabic, and English) individuals. Once the three translations were completed, one unified translation of the EDQS was created by a committee consisting of the translators and three further individuals not involved in the translation process (a sociologist and two epidemiologists). Then, the Arabic version of the EDQS was backtranslated by a native English speaker living in Morocco, who was unaware of the original English document. Once the backtranslation completed the committee reconvened to review and resolve the discrepancies between backtranslation and the original document. Finally, a pretest was conducted with a group of lay native Arabic speakers (30 subjects). Discrepancies were resolved by group consensus. Globally, the adaptation did not cause any particular problems.

At the time of the discharge, patients were approached by independent investigator who explained the purpose of the study. When patients agree to participate; they were requested to complete the EDQS anonymously. The questionnaires were recovered immediately after completion. The questionnaires were self-completed by the patients with a high education level (secondary/ higher) or administered by the same investigator if the education level was lower (none/primary). No questionnaires were taken home.

A subgroup of 66 patients was reinterviewed using the EDQS over the telephone after the initial interview to examine the test-retest reliability of the questionnaire. The first and the only version of EDQS; who estimated satisfaction with overall care, willingness to return, and reported problems of patients who were discharged from ED; was developed and validated in English. This format is easily measured allowing to confidently focusing improvement efforts [8].

Continuous data were presented as the mean ± standard deviation for variables with a normal distribution, and as the median with interquartile range (IQR) for variables with skewed distributions. For Categorical data, the percentages and number of patients in each category were presented. Data collected from patients who participated in both the initial and follow-up interviews were used to estimate the test-retest reliability of the EDQS. The test-retest reliability of the EDQS dimensions was investigated using the Cohen’s kappa. According to Landis and Koch, kappa coefficients of less than 0.0 are poor, 0.0 to 0.20 are slightly poor, 0.21 to 0.40 are fair, 0.41 to 0.60 are moderate, 0.61 to 0.80 are substantial, and 0.81 to 1.00 are almost perfect [13]. Determinants of patient satisfaction were performed using univariate and multivariate ordinal logistic regression with the proportional odds ratio. Variables with a *P* ≤ 0.20 by univariate analysis were selected for inclusion in a multivariate analysis. A two-tailed P value < 0.05 was considered significant. All statistical analyses were carried out using SPSS for Windows 13.0 (SPSS, Inc., Chicago, IL, USA).

## Results

Four hundred ninety surveys were distributed to the patients who were discharged from the ED during the 1 week study period. One hundred sixty six patients declined to participate in the study naming various reasons such as lack of time or simply unwillingness to participate in the study. Eighty nine were lost sight. Two hundred thirty five surveys were returned of which 23 surveys were incomplete (omission to fill up one item or more). This left 212 patients for the study. The questionnaires were filled up by 41,5% (n = 88) of patients themselves, while the remaining 58.5% (n = 124) were filled by the interviewer. Table 1 summarized demographic, socio-economic, and health characteristics.

**Table 1:** The demographic, socio economic and health characteristics of patients (n = 212)

**Variables**	**n (%)**
**Age (years);** mean ± SD	42.5 ± 16.2
**Gender**	
Female	94 (44.3)
Male	118 (55.7)
**Marital status**	
Single	60 (28.3)
Married	138 (65.1)
Separated	8 (3.8)
Widowed	6 (2.8)
**Residence**	
Urban	160 (75.5)
Rural	52 (24.5)
**Education Level**	
No education	102 (48.1)
Primary	38 (17.9)
Secondary	46 (21.7)
High	26 (12.3)
**Health Insurance**	
Yes	50 (23.6)
No	162 (76.4)
Priority code*	
Emergent	36 (17)
Urgent	126 (59.4)
Non-urgent	50 (23.6)
**Waiting time (per minuts);** median (IQR)	47.5 (15–100)
**Day of week**	
Weekday	178 (84)
Weekend	34 (16)
**Guidance of patients**	
Discharge	77(36.1)
Admit	118(55.2)
Transfer	14(6.3)
Not seen	3(2.4)
**Main diagnostics**	
Abdominal pain	74 (34.9)
Trauma	36 (17)
Asthma-bronchitis-emphysema	32 (15.1)
Hand laceration	28 (13.2)
Chest pain	20 (9.4)
Gastrointestinal hemorrhage	12 (5.6)
Others	10 (4.7)

*SD*, Standard deviation; *IQR*, Interquartile range; *n* (%): Number (percentage).

*Triage classification per Waldrop et al. ^10^.

Sixty six subjects were reinterviewed by the EDQS over the telephone after the initial interview. The median (range interval) between the initial and follow-up interviews was 11 days (3 to 15 days). The Cohen’s kappa values ranged from 0.81 to 0.95 (Table 2).

**Table 2:** Cohen’s kappa values, and distributions of rating of satisfaction with overall care, willingness to return, and patient-reported problems (n = 212)

**Questions**	**Cohen’s kappa value**	**n (%)**
**Satisfaction with overall care**	**0.81**	**---**
Excellent	**---**	**28 (13.2)**
Very good	**---**	**22 (10.4)**
Good	**---**	**90 (42.5)**
Fair	**---**	**44(20.8)**
Poor	**---**	**28 (13.2)**
**Willingness to return**	**0.83**	**---**
Yes	**---**	**148 (69.8)**
No	**---**	**64 (30.2)**
**Patient-reported problems**	**---**	**---**
Help not received when needed	**0.85**	**72 (34)**
Poor explanation of potential causes of problem	**0.91**	**90 (42.5)**
Not told about potential wait time	**0.86**	**168 (79.2)**
Not told when to resume normal activity	**0.92**	**92 (43.4)**
Poor explanation of test results	**0.88**	**114 (53.8)**
Not told when to return to the ED	**0.95**	**64 (30.2)**

*n* (%): Number (percentage); *ED*: Emergency Department.

The construct validity of EDQS demonstrated a powerful association between patient satisfaction with overall care and willingness to return in one hand, and between patient satisfaction with overall care and patient reported problem in the other hand (Table 3).

**Table 3:** Association between satisfaction with overall care; willingness to return, and patient-reported problems

**Questions**	**OR**	**95% CI**	***P *value**
**Willingness to return**			
Yes	**0.13**	**0.71-0.25**	**< 0.001**
No	**1**	**---**	**---**
**Patient-reported problems**			
Help not received when needed	**2.99**	**1.54-5.81**	**< 0.001**
Poor explanation of potential causes of problem	**3.01**	**1.56-6.11**	**0.001**
Not told about potential wait time	**1.99**	**1.21-4.81**	**0.002**
Not told when to resume normal activity	**1.89**	**1.14-3.14**	**0.014**
Poor explanation of test results	**2.08**	**1.26-3.45**	**0.004**
Not told when to return to the ED	**2.11**	**1.30-3.48**	**0.005**

*OR*, Odds Ratio; 95% *CI*, 95% Confidence Interval; *N*, Number. %: Percentage. 1 indicated a reference category; Odds ratio > 1 is indicated a bad satisfaction. Odds ratio < 1 is indicated a better satisfaction.

More than half of participants were satisfied with the overall care (66.1%), and two thirds will return to our unit, if they have another problem requiring emergency care (69.8%). The participants responded to the questions about the problems reported to patient satisfaction. The most common problems were about waiting time in 79.2% of patient (not told about potential wait time), and about test results in 53.8% (Poor explanation of test results). Distribution of rating of overall care, willingness to return, and specific patient-reported problems are summarized in Table 2.

Univariate analysis of the selected variables showed their relationship with the overall satisfaction (Table 4). Concerning Factors related to higher satisfaction: patients who were triaged emergent and urgent were more satisfied compared to non-urgent patients. Waiting time < 15 min was also related to higher satisfaction with the ED. Concerning Factors related to lesser satisfaction in univariate analysis, two variables were noted: Distance patient’s home hospital ≤ 10 Kilometers, and weekday’s admissions.

**Table 4:** Univariate and multivariate analysis of predictors of satisfaction related to demographics, socioeconomics, and health characteristics

**Characteristics**	**Univariate analysis**			**Multivariate analysis**		
	**OR**	**95% CI**	***P *value**	**OR**	**95% CI**	***P *value**
**Age (per years)**	**1.01**	**0.99-1.02**	**0.6**	**---**	**---**	**---**
**Gender**						
Male	**1**	**---**	**---**	**---**	**---**	**---**
Women	**0.93**	**0.56-0.52**	**0.78**	**---**	**---**	**---**
**Marital status**						
Married	**1**	**---**	**---**	**1**	**---**	**---**
Others	**0.52**	**0.31-0.86**	**0.10**	**0.67**	**0.34-1.16**	**0.15**
**Residence**						
Urban	**1**	**---**	**---**	**---**	**---**	**---**
Rural	**0.81**	**0.47-1.40**	**0.33**	**---**	**---**	**---**
**Distance patient’s home hospital**						
≤ 10 Kilometers	**2.32**	**1.40-3.84**	**0.001**	**2.64**	**1.53-4.53**	**< 0.001**
> 10 Kilometers	**1**	**---**	**---**	**1**	**---**	**---**
**Education level**						
No education	**1.10**	**0.45-2.44**	**0.82**	**2.53**	**1.02-6.3**	**0.04**
Primary	**1.22**	**0.49-3.06**	**0.67**	**3.04**	**1.10-8.04**	**0.03**
Secondary	**2.21**	**0.91-5.38**	**0.08**	**5.19**	**2.04-3.21**	**0.001**
High	**1**	**---**	**---**	**1**	**---**	**---**
**Monthly income**						
Less than 180 euro	**0.62**	**0.38-1.03**	**0.065**	**0.78**	**0.45-1.35**	**0.37**
More than 180 euro	**1**	**---**	**---**	**1**	**---**	**---**
**Health insurance status**						
Yes	**1**	**---**	**---**	**---**	**---**	**---**
No	**1.09**	**0.5-1.98**	**0.8**	**---**	**---**	**---**
**Type of admission**						
Emergent	**0.11**	**0.05-2.26**	**<0.001**	**0.15**	**0.04-0.31**	**< 0.001**
Urgent	**0.26**	**0.13-0.52**	**<0.001**	**0.35**	**0.15-0.86**	**0.02**
Non urgent	**1**	**---**	**---**	**1**	**---**	**---**
**Waiting time**						
>15 minuts	**1**	**---**	**---**	**1**	**---**	**---**
≤15 minuts	**0.41**	**0.24-0.71**	**0.001**	**0.41**	**0.23-0.75**	**0.003**
**Day of week**						
Weekday	**1.97**	**1.03-3.74**	**0.04**	**2.66**	**1.32-5.34**	**0.006**
Weekend	**1**	**---**	**---**	**1**	**---**	**---**

*OR*, Odds Ratio; 95% *CI*, 95% Confidence Interval; *N*, Number. %: Percentage. 1 indicated a reference category; Odds ratio > 1 is indicated a bad satisfaction. Odds ratio < 1 is indicated a better satisfaction.

We also studied the effect of the previous variables on the satisfaction after adjustment by variables in a multivariate model. Concerning factors related to higher satisfaction in multivariate analysis, two independent variables were identified: type of admission; with patients who were triaged emergent, or urgent were more satisfied compared to non urgent; and waiting time less than 15 min in emergency department. Concerning Factors related to lesser satisfaction in multivariate analysis, three independent variables were noted: distance patient’s home hospital ≤ 10 Kilometers, weekday’s admissions, and educational level. Table 4 presents the ordinal logistic regression results.

## Discussion

This study reports the results of the first Moroccan study concerning the patient satisfaction with emergency care using the Arabic version of the EDQS. The psychometric properties of Arabic version of the EDQS showed excellent reliability and validity.

The ED is a unique department among other medical care services, thus, understanding the factors affecting patient satisfaction is essential [14].

Our study, like similar studies, indicates that the general satisfaction of clients is high, although there are many unmet needs [8,15-17]. More than half of participants (66.1%) were satisfied with the overall care, and rated the care received in the emergency department “Excellent”, “Very Good”, or “Good”, and, 69.8% of patients will return to our unit, if they have another problem requiring emergency care.

Our funding also indicated that there is an association between satisfaction and type of emergency admission, waiting time less than 15 min, weekday’s admissions, educational level, and distance patient’s home hospital ≤10 Kilometers.

In this study, "Emergent" and “Urgent” patients perceived their throughput times more favorably than non urgent as showed in a previous study [18]. More acute patients may be more satisfied with their ED care precisely because they receive greater interpersonal attention from ED providers or get seen faster than those who are less acute [19]. The finding of our study revealed that average time a patient waited to be seen by an emergency physician was 47.5 min. Compared with similar studies, the waiting time in our study was much more [20-23]. This lengthy waiting time (more than 15 min) had a direct relationship with patient dissatisfaction in emergency department. This feature has been reported by many other studies whose included both the real stay-in-the-waiting-room time and the waiting time perceived by the patient [20-23], the latter was more predictive of satisfaction than the former [20,21].

The day of admission influenced patient satisfaction during this study with lesser satisfaction associated to weekday’s admissions. This is probably due to the greater number of consultations and emergencies. Patient who consulted the week-end are generally seriously ill, and therefore can be more satisfied because of the quick management.

The most consistent variables associated with a lesser satisfaction were; distance patient’s home hospital ≤10 Kilometers. The management conditions in our hospital are supposed to be better than in rural or urban hospitals located close to Rabat. However, inhabitant patient within 10 kilometers distance from hospital can consult for every health problem, they know the real situation, and they hope more. Thus, patient satisfaction was probably influenced by the discrepancy between patient’s hopes on quality of care and the real quality of the medical management offered. More their hopes are close to emergency unit realities, more satisfied will be the patients.

Patients with a lower educational level (illiterate, primary, and secondary levels) were less satisfied. Patients with a high educational level listen and integrate medical debate. Thus, they accept that their rescue was dependent on good management, despite uncomfortable conditions.

Some specific problems have been reported. More than half of patient claims about the potential waiting time, and explanation of test results. These two criteria reflect more the expectations of the patient in the acute phase. Communication and the way to explain all the exits were believed to be more important medical and paramedical than technical skills. [8-11,14-16,18-21,24]. However, others information (subsequent activity, potential causes of problem, when to return to the ED) does not seem to be a priority. Indeed, patients wanted to understand what is happening, and they have fewer worries about what will happen in the future. This may be attributed to cultural specificities of Moroccan people. The Moroccan population is Muslim. The certainty that fate is under the control of God, and therefore the well-being and disease, is undeniable. The patients were using prayer to help break the stressful situations they are subjected, and win the blessing of god. The disease is considered a predestined fact and should not be directly linked to a possible failure in the management of medical and paramedical.

### Limitations

There are some limitations in this study, some of which are inherent in the survey’s methodology. First, EDQS is a self- or interviewer-administered questionnaire. So, patients with no or a low educational level filled up the questionnaire with the help of the interviewer, inducing thus potential bias. They may be influenced by characteristics and attitudes of the interviewer, particularly in face to face situations [25]. The alternative would be to exclude low-literacy participants. However, the decision to include these participants was more important than the risk of bias, this group of participants was a better representation of a Moroccan population. Furthermore, the different data collection methods (self-administration and administration by an investigator) have advantages and disadvantages; however, no consensus is available concerning modalities of administering questionnaires in low-literacy populations [25]. Second, the study was conducted in one site; thus, the results cannot be generalized to all Moroccan Hospitals. Third, we studied patients’ satisfaction for 1 week of the year. The patients admitted during other seasons could differ from our sample. Fourth, the sample size was relatively small. Finally, the staff was not blinded that a study was done on patient satisfaction.

## Conclusion

In summary, slightly more than half of participants were satisfied with the overall care, and two thirds will return to our unit, if they have another problem requiring emergency care. The predictive factors objected in this view were; educational level, waiting time, emergency degree, and week of admission. These data underline cultural specificities of Moroccan population, which suggested the need for the medical staff to consider the different interactions between those predictive factors in order to develop some supportive tools like the welcome handbook. For emergency departments to remain profitable, it will be more important than ever before to meet the needs and expectations of their current and potential users. This can be accomplished by a program designed to reduce cost and waiting time and improve communication, and by other programs to educate the user so that the user's expectations more closely conform with what is actually needed or can be economically provided.

Theories of human behaviors may offer useful means of understanding factors such as motivation and designing strategies to change practice. Whatever the level of development of a country, the importance of a patient-centered approach is now widely recognized. It is difficult to state with certitude what will lead to improved patient satisfaction in the ED; however, a few research-based suggestions can be made.

## Competing interests

The authors declare that they have no competing interests.

## Authors’ contributions

DN and BJ participated equally to the work. DN participated in the design of the study, and in the acquisition of data. BJ draft the manuscript. AB, ZA MN, KA, and MD participated in the acquisition of data. BBA, and ZAA participated in the coordination of data. AR conceived of the study, participated in the design of the study, performed the statistical analysis and interpretation of data, and gave the final approval of the manuscript. All authors read and approved the final manuscript.

## References

[B1] Eric R Messner MS Quality of care and patient satisfaction the improvement efforts of one emergency department Top Emerg Med 2005 27 132 41

[B2] Razzak JA Kellermann AL Emergency medical care in developing countries: is it worthwhile? Bull World Health Organ 2002 80 900 5 12481213 PMC2567674

[B3] Mock C Quansah R Krishnan R Arreola-Risa C Rivara F Strengthening the prevention and care of injuries worldwide Lancet 2004 363 2172 9 10.1016/S0140-6736(04)16510-0 15220042

[B4] Kobusingye OC Hyder AA Bishai D Hicks ER Mock C Joshipura M Emergency medical systems in low-and middle-income countries: recommendations for action Bull World Health Organ 2005 83 626 31 16184282 PMC2626309

[B5] Collecte CDIM World Health Organization: Country Cooperation Strategy for WHO and Morocco 2004–2007 http://www.who.int/countries/en/cooperation_strategy_mar_en.pdf

[B6] Sun BC Adams J Orav EJ Rucker DW Brennan TA Burstin HR Determinants of patient satisfaction and willingness to return with emergency care Ann Emerg Med 2000 35 426 34 10783404

[B7] Sun BC Adams JG Burstin HR Validating a model of patient satisfaction with emergency care Ann Emerg Med 2001 38 527 32 10.1067/mem.2001.119250 11679864

[B8] Soufi G Belayachi J Himmich S Ahid S Soufi M Zekraoui A Abouqal R Patient satisfaction in an acute medicine department in Morocco BMC Health Serv Res 2010 10 149 10.1186/1472-6963-10-149 20525170 PMC2900260

[B9] Burstin HR Swartz K O’Neil AC Orav EJ Brennan TA The effect of change of health insurance on access to care Inquiry 1998 35 389 97 10047769

[B10] Waldrop R Harper D Mandry C Prospective assessment of triage in an urban emergency department South Med J 1997 90 1208 12 10.1097/00007611-199712000-00009 9404907

[B11] Choi YF Wong TW Lau CC Triage rapid initial assessment by doctor (TRIAD) improves waiting time and processing time of the emergency department Emerg Med J 2006 23 262 5 10.1136/emj.2005.025254 16549569 PMC2579496

[B12] Beaton DE Bombardier C Guillemin F Ferraz MB Guidelines for the process of cross cultural adaptation of self-report measures Spine 2000 25 3186 91 10.1097/00007632-200012150-00014 11124735

[B13] Ladis JR Koch GG The measurement of observer agreement for categorical data Biometrics 1977 33 159 74 10.2307/2529310 843571

[B14] Hall MF Press I Keys to patient satisfaction in the emergency department: results of a multiple facility study Hospital Health Service Adm 1996 41 515 32 10162399

[B15] Bleich SN Özaltin E Murray CJL How does satisfaction with the health-care system relate to patient experience? Bull World Health Organ 2009 87 271 8 10.2471/BLT.07.050401 19551235 PMC2672587

[B16] Boudreaux ED Ary RD Mandry CV Determinants of patient satisfaction in a large municipal ED: The role of demographic variables, visit characteristics, and patient perceptions Am J Emerg Med 2000 18 394 400 10919526 10.1053/ajem.2000.7316

[B17] Brédart A Mignot V Rousseau A Dolbeault S Beauloye N Adam V Elie C Léonard I Asselain B Conroy T Validation of the EORTC QLQ-SAT32 cancer inpatient satisfaction questionnaire by selfversus interview-assessment comparison Patient Educ Couns 2004 54 207 212 10.1016/S0738-3991(03)00210-6 15288916

[B18] Boudreaux ED Friedman J Chansky ME Baumann BM Emergency department patient satisfaction: examining the role of acuity Acad Emerg Med 2004 11 162 8 14759959

[B19] Boudreaux ED O’Hea EL Patient satisfaction in the Emergency Department: a review of the literature and implications for practice J Emerg Med 2004 26 13 26 10.1016/j.jemermed.2003.04.003 14751474

[B20] Nguyen Thi PL Lê TG Empereur F Briançon S Satisfaction of patients hospitalized in Ho Chi Minh City, Vietnam Sante Publique 2002 14 345 60 10.3917/spub.024.0345 12737083

[B21] Bursch B Beezy J Shaw R Emergency department satisfaction: what matters most? Ann Emerg Med 1993 22 586 91 10.1016/S0196-0644(05)81947-X 8442550

[B22] Soleimanpour H Gholipouri C Salarilak S Raoufi P Vahidi RG Rouhi AJ Ghafouri RR Soleimanpour M Emergency department patient satisfaction survey in Imam Reza Hospital, Tabriz, Iran Int J Emerg Med 2011 27;4 2 21407998 10.1186/1865-1380-1-2 PMC3051889

[B23] Hedges JR Trout A Magnusson AR Satisfied Patients Exiting the Emergency Department (SPEED) Study Acad Emerg Med 2002 9 15 21 10.1111/j.1553-2712.2002.tb01161.x 11772664

[B24] Crow R Gage H Hampson S Hart J Kimber A Storey L Thomas H The measurement of satisfaction with healthcare: implications for practice from a systematic review of the literature Health Technol Assess 2002 6 1 244 12925269 10.3310/hta6320

[B25] Damghi N Khoudri I Oualili L Abidi K Madani N Zeggwagh AA Abouqal R Measuring the satisfaction of intensive care unit patient families in Morocco: a regression tree analysis Crit Care Med 2008 36 2084 91 10.1097/CCM.0b013e31817c104e 18552683

